# Glyphosate Exposure, Oxidative Stress, Mitochondrial Dysfunction, and Mortality Risk in US Adults: Insights from the National Health and Nutrition Examination Survey

**DOI:** 10.3390/toxics13050373

**Published:** 2025-05-04

**Authors:** Yu-Wei Fang, Hsuan-Cheng Lin, Chikang Wang, Chien-Yu Lin

**Affiliations:** 1 Division of Nephrology, Department of Internal Medicine, Shin-Kong Wu Ho-Su Memorial Hospital, Taipei 111, Taiwan; m005916@ms.skh.org; 2 School of Medicine, College of Medicine, Fu Jen Catholic University, New Taipei City 242, Taiwan; 3 School of Medicine, College of Medicine, Taipei Medical University, Taipei 110, Taiwan; linxuancheng1031@gmail.com; 4 Department of Environmental Engineering and Health, Yuanpei University of Medical Technology, Hsinchu 300, Taiwan; ckwang@mail.ypu.edu.tw; 5 Department of Internal Medicine, En Chu Kong Hospital, Taipei 237, Taiwan

**Keywords:** glyphosate, glyphosate-based herbicides (GBHs), pyrazino-s-triazine derivative of 4-α-hydroxy-5-methyl-tetrahydrofolate (MeFox), methylmalonic acid (MMA), mortality, National Health and Nutrition Examination Survey (NHANES)

## Abstract

**Purpose:** Glyphosate and glyphosate-based herbicides (GBHs) are widely used across the globe. Experimental research indicates that these herbicides may elevate oxidative stress and impair mitochondrial function. However, the relationship between glyphosate exposure, oxidative stress, and mitochondrial function remains poorly characterized in epidemiological studies. In particular, the role of oxidative stress and mitochondrial function biomarkers in mediating the mortality risk associated with glyphosate exposure in nationally representative populations is not well understood. **Approach and Results:** In this study, we utilized data from the 2013–2014 National Health and Nutrition Examination Survey (NHANES), encompassing 1464 participants aged 18 years and older. This dataset was linked to mortality records from the National Center for Health Statistics (NCHS), with follow-up data extending through 2019. The primary objective was to examine the associations between urinary glyphosate levels and biomarkers of oxidative stress and mitochondrial function—specifically pyrazino-s-triazine derivative of 4-α-hydroxy-5-methyl-tetrahydrofolate (MeFox) and methylmalonic acid (MMA)—and to evaluate the role of these biomarkers in influencing glyphosate-related mortality outcomes. **Results:** Urinary glyphosate levels were positively associated with serum MMA and MeFox in weighted multiple linear regression models. For MMA, glyphosate showed significant positive associations in both adjusted models (Model 2: β = 0.061, *p* = 0.001). Similarly, urinary glyphosate was strongly associated with MeFox in all models (Model 2: β = 0.215, *p* < 0.001). During a median follow-up of 69.57 months, 116 deaths occurred, including 44 from cardiovascular causes. Glyphosate was not significantly associated with all-cause or cardiovascular mortality in the overall population. However, subgroup analysis revealed significant associations in individuals with higher MeFox levels (≥50th percentile) for all-cause mortality (HR = 1.395, *p* = 0.027) and borderline associations for cardiovascular mortality (HR = 1.367, *p* = 0.051). When adjusted for MMA, glyphosate was significantly associated with increased all-cause mortality in participants with MMA levels below the 50th percentile (HR = 2.679, *p* = 0.001), with a significant interaction between glyphosate and MMA for all-cause (*p* = 0.002) and cardiovascular mortality (*p* = 0.038). **Conclusions:** In this comprehensive analysis of NHANES data, urinary glyphosate levels were associated with biomarkers of oxidative stress and mitochondrial function. While no overall mortality associations were observed, glyphosate exposure was linked to increased all-cause mortality in subgroups with lower MMA or higher MeFox levels. These findings highlight the role of oxidative stress and mitochondrial function in glyphosate-related health risks and the need for further research to identify vulnerable populations.

## 1. Introduction

Glyphosate and glyphosate-based herbicides (GBHs), first introduced commercially in 1974, have become the most widely used herbicides globally, owing to their broad-spectrum efficacy and reputation for safety [1]. Their effectiveness stems from their unique mechanism of action, which targets 5-enolpyruvylshikimate-3-phosphate synthase in the shikimate pathway, critical for plant growth [1]. The adoption of glyphosate-resistant transgenic crops in 1996 significantly increased the use of glyphosate and GBHs, with nearly 90% of such crops engineered for glyphosate resistance [2]. Despite their widespread adoption, concerns have arisen over glyphosate’s environmental and health impacts. They have been shown to contaminate water sources and infiltrate the food chain [3]. In 2015, the World Health Organization classified glyphosate as a Group 2A carcinogen, which heightened concerns about its safety [3]. In addition to its potential link to cancer, recent research has connected glyphosate exposure in the general population to a range of health problems, such as anemia, disruptions in iron homeostasis, diabetes, metabolic syndrome, and cognitive impairment [4,5,6,7]. Furthermore, a nationwide study found a correlation between urinary glyphosate concentrations and a heightened risk of death [8].

Experimental studies have consistently demonstrated that glyphosate and GBHs increase oxidative stress across various cell types [9,10,11]. They are also known to impair mitochondrial function, including disruptions in mitochondrial membrane potential and oxidative phosphorylation [12,13,14]. Epidemiological studies have identified positive associations between glyphosate exposure and oxidative stress biomarkers, such as lipid peroxidation markers (e.g., 8-iso-prostaglandin-F2α and malondialdehyde) and DNA oxidation markers [15,16,17,18]. However, no study to date has comprehensively examined the relationship between glyphosate exposure and a broad spectrum of mitochondrial function biomarkers. Methylmalonic acid (MMA) is a key biomarker that reflects the proper functioning of the Krebs cycle, a crucial pathway within mitochondria [19]. Since mitochondrial dysfunction often leads to increased oxidative stress [20], elevated MMA levels indicate both impaired mitochondrial function and increased oxidative stress. Additionally, MeFox (pyrazino-s-triazine derivative of 4-α-hydroxy-5-methyl-tetrahydrofolate), a stable oxidation product of 5-methyltetrahydrofolate (5-MTHF), is considered a biomarker of folate metabolism and oxidative stress [21,22]. Given the intricate interplay between one-carbon metabolism, oxidative stress, and mitochondrial function [23], MeFox levels may also indirectly reflect mitochondrial dysfunction. Elevated MMA and MeFox levels have been associated with an increased risk of various chronic diseases in epidemiological studies [24,25,26,27]. However, no epidemiological studies to date have explored the relationship between glyphosate exposure, MMA, and MeFox or the role of these biomarkers in connecting glyphosate exposure to mortality risk.

To address this gap in knowledge, we utilized the 2013–2014 National Health and Nutrition Examination Survey (NHANES), a unique dataset that includes comprehensive measurements of urinary glyphosate, serum MMA, and serum MeFox, alongside mortality outcomes tracked by the National Center for Health Statistics (NCHS) through 2019. This rich dataset provided a valuable opportunity to investigate the interrelationships between glyphosate exposure, biomarkers of oxidative stress and mitochondrial function (represented by MeFox and MMA), and mortality risk in a nationally representative sample of U.S. adults. Our study aimed to advance the understanding of how glyphosate exposure interacts with key biological pathways—oxidative stress and mitochondrial function—and their potential roles in shaping mortality risks.

## 2. Materials and Methods

### 2.1. Study Population

**Figure 1:**
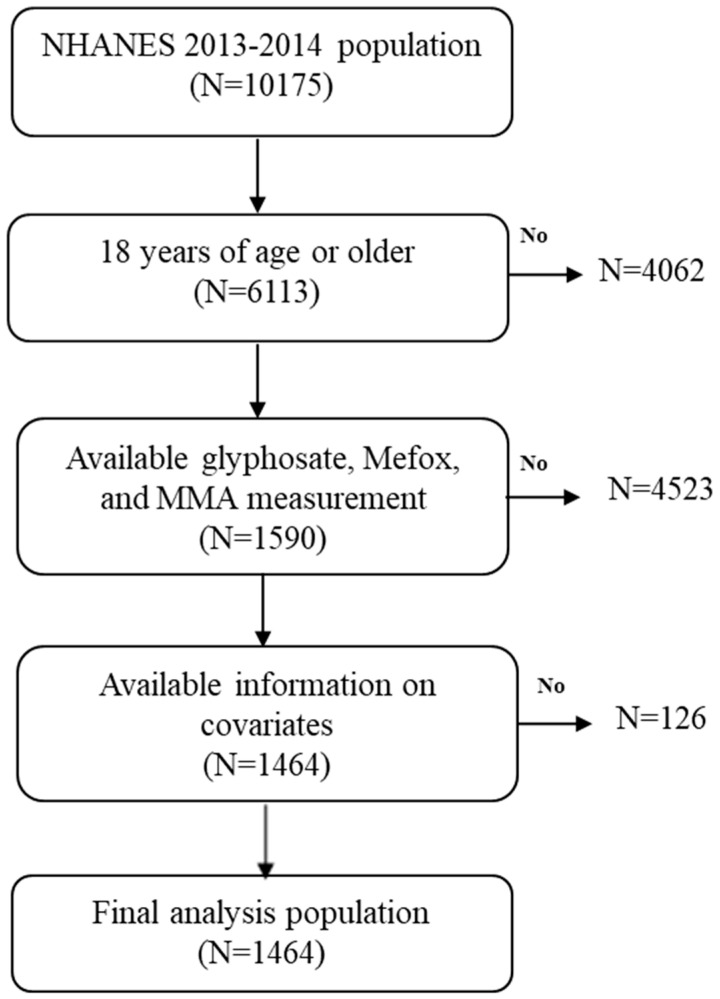
Flow chart algorithm.

The NHANES, a biennial survey, provides a comprehensive overview of the health and nutritional status of the U.S. population. Detailed information regarding survey methodology and consent procedures is available on the NHANES website [28]. This study utilized data from the NHANES 2013–2014 cycle, which initially included 10,175 participants. Of these, 6113 were aged 18 years or older. A total of 4523 participants were excluded due to missing data on urinary glyphosate, serum MeFox, or serum MMA. Among the remaining 1590 eligible individuals, 126 were further excluded due to incomplete covariate information. The final analytic sample consisted of 1464 adults. The participant selection process is illustrated in Figure 1.

### 2.2. Measurement of Urinary Glyphosate Levels

For participants aged 19 years and older, the NHANES 2013–2014 survey assessed urinary glyphosate concentrations in a one-third subsample of participants. This study utilized data from glyphosate levels, which were determined using liquid chromatography coupled to tandem mass spectrometry (LC-MS/MS). Analyses followed the Clinical Laboratory Improvement Amendments guidelines to ensure accuracy. The limit of detection (LOD) of glyphosate was 0.2 ng/mL. For concentrations below LOD, a value of LOD/√2 was imputed, following the NHANES protocol. Detailed methodologies for glyphosate analysis are available on the NHANES website [29].

### 2.3. Measurement of Serum MeFox

For participants aged 19 years and older, the NHANES 2013–2014 survey assessed serum MeFox in all participants. Serum MeFox was measured using the LC-MS/MS method. MeFox was separated under isocratic mobile phase conditions within six minutes and quantified via LC-MS/MS. The LOD of MeFox was 0.08 nmol/L. For concentrations below LOD, a value of LOD/√2 was imputed. Detailed methodologies for MeFox analysis are available on the NHANES website [30].

### 2.4. Measurement of Serum MMA

The NHANES 2013–2014 survey assessed serum MMA in all participants in participants aged 19 years and older. MMA was analyzed using LC-MS/MS after derivatization to its dibutylester. Serum samples were extracted using liquid–liquid extraction, followed by derivatization with butanol. The resulting dibutylester was then analyzed by LC-MS/MS using multiple reaction monitoring. Chromatographic separation was achieved within 5.9 min using isocratic mobile phase conditions, ensuring separation from isobaric succinic acid. The LOD of MMA was 22.1 nmol/L. For values below the LOD, a value of LOD/√2 was imputed. Detailed methodologies for MMA analysis are available on the NHANES website [31].

### 2.5. Covariates

Data from the NHANES database were utilized, encompassing a range of variables. Sociodemographic factors were included alongside smoking status, classified as active smokers, individuals exposed to environmental tobacco smoke (ETS), and non-smokers. Alcohol consumption was categorized based on whether participants reported consuming at least 12 alcoholic drinks in the past year. Other variables included the body mass index (BMI) and physical activity levels, which were measured using metabolic equivalent scores and grouped into tertiles [32]. Hypertension was defined as blood pressure readings ≥ 140/90 mmHg or the use of antihypertensive medication. Diabetes was identified based on fasting serum glucose levels ≥ 126 mg/dL, glycated hemoglobin levels ≥ 6.5%, or the use of diabetes medication. Hypercholesterolemia was determined by low-density lipoprotein cholesterol levels ≥ 130 mg/dL or the use of cholesterol-lowering medication. Chronic kidney disease was defined as an estimated glomerular filtration rate of < 60 mL/min/1.73 m^2^ [33]. Serum vitamin B12 levels were measured using the Elecsys Vitamin B12 assay, an automated test that forms a complex with intrinsic factor and biotin-labeled vitamin B12. The resulting chemiluminescent signal was measured to determine vitamin B12 concentration based on a calibration curve [34]. Urinary creatinine was analyzed as an independent variable to adjust for variations in urine concentration.

### 2.6. Outcomes

The NCHS has linked the 2013–2014 NHANES data to national mortality records, allowing for long-term follow-up of participant health outcomes. This linkage provides comprehensive data on all-cause and cause-specific mortality through 2019 for participants aged 18 and older. For this study, we utilized data on participant survival status and follow-up duration. Given the relatively short follow-up period and limited number of observed deaths, our analysis focused on all-cause and cardiovascular mortality. A detailed description of the analytical methods employed in this study is available on the NCHS website [35].

### 2.7. Statistics

The data analysis involved calculating the geometric mean and geometric standard error of urinary glyphosate, MeFox, and MMA across various population subgroups. To evaluate distinctions among subgroups, we utilized two statistical approaches: two-tailed Student’s *t*-tests for comparing pairs of groups and one-way analysis of variance for analyzing differences across multiple groups. Natural logarithm (ln) transformations were applied to glyphosate, MeFox, MMA, urinary creatinine, and serum vitamin B12 due to their skewed distributions. To ensure population representativeness and obtain valid estimates and standard errors, analyses accounted for the NHANES’s complex multistage sampling design by incorporating examination weights, primary sampling units, and strata, in accordance with the NHANES analytic guidelines [36]. Linear regression analyses with complex sampling were conducted to explore associations between urinary glyphosate, MeFox, and MMA. Two regression models were employed to adjust for confounders. Model 1 accounted for age, sex, race/ethnicity, the poverty-to-income ratio, the BMI, urinary creatinine, smoking status, alcohol use, physical activity levels, and serum vitamin B12 levels. Model 2 included all variables from Model 1 and further adjusted for hypertension, diabetes mellitus, chronic kidney disease, and hypercholesterolemia. Only associations that remained statistically significant in both models were deemed robust, demonstrating their stability despite the inclusion of additional confounders. To assess the dose–response relationship, we divided urinary glyphosate into quartiles and applied a complex-samples general linear model (Model 2 covariates) to obtain adjusted least-square means for ln-MeFox and ln-MMA; these were back-transformed to geometric means ± SE, and trend *p*-values were derived by modelling quartile medians as an ordinal term. We also produced weighted scatter-plot regressions for ln-urinary glyphosate versus ln-serum MeFox and ln-serum MMA, using the same covariate set as Model 2.

Mortality hazard ratios (HRs) were evaluated per unit increase in urinary glyphosate. The analysis utilized weighted Cox proportional hazards regression, adjusting for covariates defined in either Model 3 (Model 2 plus MeFox) or Model 4 (Model 2 plus MMA) to examine the influence of MeFox or MMA on glyphosate-related all-cause mortality. To evaluate effect modification, we dichotomized serum MeFox and serum MMA separately. Weighted Cox proportional-hazards models were then fitted within each stratum (MeFox < median vs. ≥ median; MMA < median vs. ≥ median). An interaction term (ln-glyphosate × biomarker stratum) was tested in the overall cohort to assess statistical interaction. Statistical analyses were performed using SPSS version 20 (SPSS Inc., Chicago, IL, USA), with statistical significance set at a *p*-value of 0.05.

## 3. Results

The study participants had an average age of 48.64 years (±17.75), with ages spanning from 19 to 80 years. Glyphosate was detectable in 79.8% of individuals, with a mean concentration of 0.54 µg/L (±0.53) and a range of 0.14 to 6.46 µg/L. The average serum MeFox level was 2.09 nmol/L (±1.85), ranging from 0.17 to 22.90 nmol/L. Additionally, the mean serum MMA concentration was 174.12 nmol/L (±132.55), with values varying between 26.7 and 1610.00 nmol/L.

Table 1 presents the geometric means of glyphosate, MeFox, and MMA among different subgroups. Females and older adults exhibited higher glyphosate levels (adjusted for urinary creatinine), while both MeFox and MMA increased with age. Ethnicity significantly influenced all biomarkers, with non-Hispanic White people showing the highest levels of glyphosate, MeFox, and MMA. Participants with hypertension, diabetes, and hypercholesterolemia had elevated levels of all three biomarkers, while those with chronic kidney disease had significantly higher MeFox and MMA levels. Smoking status and the BMI showed minimal associations, with non-smokers exhibiting higher levels of both urinary glyphosate and MeFox, while individuals with a higher BMI had increased MeFox levels.

**Figure 2:**
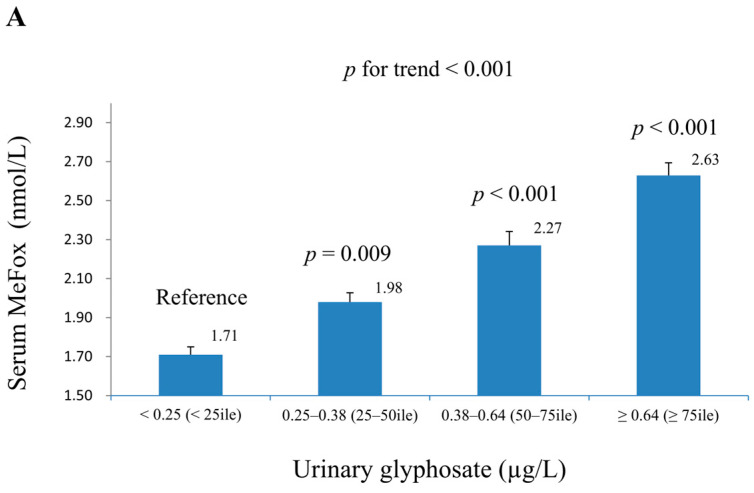

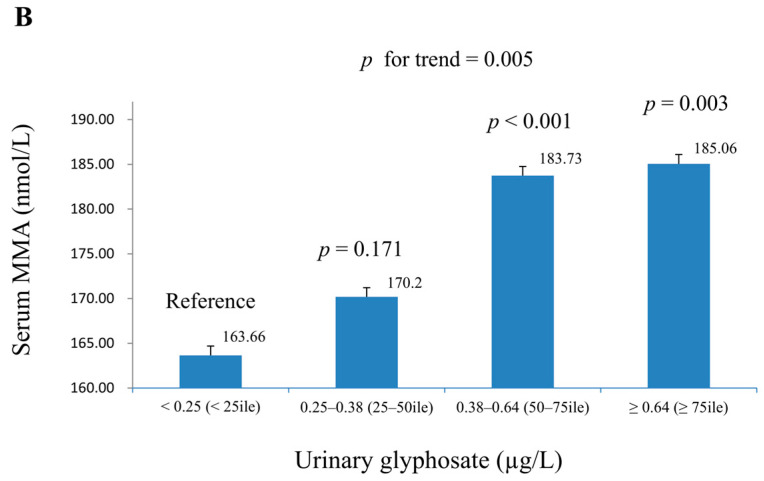
The geometric mean (geometric SE) of serum MeFox and MMA levels across quartiles of urinary glyphosate based on multiple linear regression models (adjusted for Model 2), with the results weighted to account for the sampling strategy (N = 1464). (**A**): Serum MeFox. (**B**): Serum MMA. Comparisons are made against the lowest quartile of urinary glyphosate.

Table 2 summarizes the associations of ln-MMA and ln-MeFox with continuous covariates in weighted multiple linear regression models. For ln-MMA, urinary glyphosate showed a significant positive association in both Model 1 (β = 0.063, *p* = 0.001) and Model 2 (β = 0.061, *p* = 0.001). Ln-MeFox also had a significant positive association in both models (Model 1: β = 0.152, *p* < 0.001; Model 2: β = 0.131, *p* < 0.001). Age was positively associated with ln-MMA (*p* < 0.001), while the poverty–income ratio and ln-vitamin B12 levels showed significant negative associations (*p* ≤ 0.001). The BMI emerged as a significant covariate in the relationship between ln-MeFox and ln-MMA in the final model (*p* = 0.049). For ln-MeFox, urinary glyphosate demonstrated a strong positive association in both Model 1 (β = 0.221, *p* < 0.001) and Model 2 (β = 0.215, *p* < 0.001). The BMI was positively associated with ln-MeFox (*p* < 0.01), while urinary creatinine and the BMI were also significant covariates in both models. Supplemental Figure S1 corroborates these findings: weighted linear regression demonstrates significant positive associations between ln-urinary glyphosate and both ln-MeFox (adjusted R^2^ = 0.23) and ln-MMA (adjusted R^2^ = 0.06). Figure 2 demonstrates the geometric mean (SE) of serum MeFox and MMA levels across quartiles of urinary glyphosate in complex multiple linear regression models (adjusted for Model 2). The geometric mean of serum MeFox increased monotonically across weighted quartiles of urinary glyphosate (Q1→Q4: 1.71→2.63 nmol/L; P-for-trend < 0.001). A similar finding was seen for serum MMA (Q1→Q4: 163.66→185.06 nmol/L; P-for-trend < 0.001).

Table 3 presents the associations of ln-MMA and ln-MeFox with ln-urinary glyphosate across various subpopulations, adjusted for multiple covariates and weighted for the sampling strategy. For ln-MeFox, significant positive associations with ln-urinary glyphosate were observed across all subgroups, with no significant interactions across subgroups. Similarly, ln-MMA showed significant positive associations with ln-urinary glyphosate in most subgroups. Notably, associations were generally weaker in individuals with a higher BMI, chronic kidney disease, or diabetes. Additionally, ethnicity revealed a significant interaction (*p* = 0.010).

Among the 1464 participants, one participant was missing outcome information. During a median follow-up of 69.57 months, 116 deaths occurred, including 44 cardiovascular deaths. Table 4 summarizes the HR for all-cause and cardiovascular mortality associated with a unit increase in ln-glyphosate across subgroups in weighted Cox regression models. After further adjustment for MeFox in Model 3, ln-glyphosate displayed a non-significant positive association with all-cause mortality. Subgroup analysis revealed a significant association in participants with MeFox levels in the ≥50th percentile (HR = 1.395, 95% CI: 1.044–1.864, *p* = 0.027). For cardiovascular mortality, ln-glyphosate was not significantly associated in the overall population. However, subgroup analysis in Model 3 indicated a borderline association among participants with MeFox levels in the ≥50th percentile (HR = 1.367, 95% CI: 0.998–1.872, *p* = 0.051). The interaction between glyphosate and MeFox on mortality outcomes was not statistically significant. When further adjusted for MMA in Model 4, ln-glyphosate was significantly associated with increased all-cause mortality exclusively in participants with MMA levels below the 50th percentile (HR = 2.679, 95% CI: 1.603–4.475, *p* = 0.001). In Model 4, no significant associations were found across MMA subgroups for cardiovascular mortality. Nevertheless, a significant interaction was identified between glyphosate and MMA on mortality outcomes (P for interaction = 0.002 for all-cause mortality; P for interaction = 0.038 for cardiovascular mortality). Joint stratification by MeFox and MMA is summarized in Supplemental Table S1. Using the subgroup with both biomarkers below the median as the reference, a one-unit increase in ln–glyphosate was associated with a HR for all-cause mortality of 1.04 (95% CI: 0.32–3.37) in the high-MeFox/low-MMA group, 1.47 (95% CI: 0.62–3.46) in the low-MeFox/high-MMA group, and 1.05 (95% CI: 0.53–2.07) in the dual-high group; none reached statistical significance and the overall test for trend was null (*p* = 0.58). Estimates for cardiovascular mortality were likewise imprecise, with all 95% CIs spanning unity.

## 4. Discussion

Our study analyzed a nationally representative sample of adults from the U.S. and is the first to report a positive correlation between urinary glyphosate levels, MeFox, and MMA. Additionally, our findings reveal that glyphosate exposure is significantly associated with increased all-cause mortality among individuals with elevated MeFox levels and reduced MMA levels. A significant interaction between glyphosate and MMA on mortality outcomes was also observed. If these correlations suggest causation, they raise important concerns about the potential impact of glyphosate exposure on folate metabolism, oxidative stress, and mitochondrial function, as well as their combined influence on mortality risk. Given these findings, there is an urgent need for further research to investigate the underlying mechanisms, as well as their broader implications for public health in the general U.S. population.

Folate metabolism plays a critical role in the methylation cycle, which is essential for DNA synthesis and amino acid metabolism. 5-MTHF, a key component of this cycle, facilitates the conversion of homocysteine to methionine through a vitamin B12-dependent reaction [21,22]. This one-carbon metabolism not only supports the synthesis of purines, pyrimidines, and methionine but also contributes to mitochondrial function by providing essential methyl groups [37]. Under oxidative stress, 5-MTHF can be oxidized into MeFox due to elevated reactive oxygen species [38]. This oxidative disruption impairs folate metabolism, compromising mitochondrial DNA maintenance and oxidative phosphorylation [21,22]. Furthermore, the interplay between one-carbon metabolism and mitochondrial function—critical for processes such as nucleotide synthesis and methylation—highlights their interconnected nature [23]. In this context, MeFox levels may also serve as an indirect indicator of mitochondrial dysfunction. Epidemiological studies have shown a positive correlation between MeFox levels and hypertension, chronic kidney disease, as well as an increased risk of mortality [26,27,39,40].

Methylmalonyl-CoA is a crucial intermediate in the metabolism of certain amino acids (like valine, isoleucine, and methionine) and fatty acids. It is converted to succinyl-CoA by the enzyme methylmalonyl-CoA mutase (MCM), a critical step in the Krebs cycle, which is essential for cellular energy production [41]. The activity of MCM relies on vitamin B12 as an essential cofactor. Additionally, conditions such as mitochondrial dysfunction, oxidative stress, or inadequate ATP levels can indirectly impair MCM function by destabilizing the cellular environment required for its optimal activity [42,43]. When MCM activity is impaired, methylmalonyl-CoA accumulates and is hydrolyzed into MMA, leading to elevated levels. This makes MMA a valuable biomarker for assessing vitamin B12 status, oxidative stress, and mitochondrial health [19]. Elevated MMA has been linked to the progression and prognosis of chronic conditions, including cardiovascular events, renal insufficiency, cognitive decline, and cancer [19,24,25,44]. Our study revealed a positive correlation between MeFox and MMA. Given that vitamin B12 is essential for both folate metabolism and the activity of MCM, vitamin B12 levels could potentially influence this correlation. However, as we controlled for serum vitamin B12 in our analyses, a direct link to vitamin B12 deficiency is less likely. Alternatively, oxidative stress may simultaneously increase MeFox levels through folate oxidation and elevate MMA levels. This association may be attributed to their shared involvement in one-carbon metabolism and mitochondrial function.

Research suggests that glyphosate and GBHs can increase oxidative stress and negatively impact mitochondrial function. In experimental studies, glyphosate exposure has been shown to reduce ATP production and alter mitochondrial metabolism in human prostate cells [12]. Similarly, low-dose glyphosate exposure inhibits testosterone synthesis in mouse Leydig cells by inducing overproduction of mitochondrial reactive oxygen species, which leads to mitochondrial fragmentation [45]. In zebrafish brains, GBH exposure resulted in decreased mitochondrial complex activity, increased reactive species production, and mitochondrial membrane hyperpolarization [13]. A laboratory-based study using sperm samples from 66 healthy men found that exposure to GBHs at a concentration of 1 mg/L significantly reduced sperm motility and impaired mitochondrial function [46].

Several epidemiological studies have investigated the relationship between glyphosate exposure and biomarkers of oxidative stress. Occupational research suggests that farmers exposed to glyphosate may exhibit elevated levels of 8-hydroxy-2′-deoxyguanosine and malondialdehyde [15,16]. A study of 227 pregnant women in the U.S. investigated the relationship between urinary levels of glyphosate, its metabolite aminomethylphosphonic acid (AMPA), and oxidative stress biomarkers. The findings revealed an association between elevated urinary AMPA levels and increased levels of 8-iso-prostaglandin-F2α [17]. Another study of 128 infertile French men found a positive correlation between glyphosate exposure and levels of 8-hydroxy-2′-deoxyguanosine [18]. However, these studies have largely focused on specific populations and have been limited by relatively small sample sizes. Notably, none have investigated the relationship between glyphosate exposure and biomarkers of mitochondrial function. In our study, we identified a positive correlation between urinary glyphosate levels, MeFox, and MMA in a representative sample of American adults. Glyphosate-induced oxidative stress generates reactive oxygen species, which can oxidize 5-MTHF to MeFox, depleting active folate reserves. This folate depletion may disrupt downstream processes, such as DNA synthesis, further impairing mitochondrial function and contributing to the accumulation of MMA [23]. Additionally, mitochondrial dysfunction resulting from glyphosate exposure can intensify oxidative stress, creating a feedback loop that amplifies the production of both MeFox and MMA [47].

Several studies have linked glyphosate exposure to increased mortality risk. For example, research in Washington State found a 33% higher risk of Parkinson’s disease-related death among individuals residing in areas with glyphosate-associated land use [48]. Additionally, an ecological study in Argentina observed elevated cancer incidence, prevalence, and mortality rates in a town with high glyphosate pollution [49]. Consistent with these findings, previous studies using the NHANES 2013–2016 and 2013–2018 databases have demonstrated a significant association between glyphosate exposure and an increased risk of all-cause mortality [8,50]. Our analysis using data from the same cohort but with a slightly different timeframe (2013–2014) did not yield similar results. This discrepancy may be due to the smaller sample size in our study.

The association between glyphosate exposure and mortality risk was notably stronger in individuals with above-average serum MeFox levels and below-average MMA levels. MeFox elevation reflects an oxidative-stress-prone environment, even in the presence of intact mitochondrial function [38]. Elevated MeFox levels could impose a physiological burden, potentially intensifying the toxic effects of glyphosate and amplifying its impact on mortality risk. In contrast, MMA accumulates when the activity of the vitamin B₁₂-dependent enzyme MCM is chronically impaired, often due to vitamin B₁₂ deficiency or longstanding mitochondrial damage [19]. Our finding that glyphosate-related mortality was most pronounced in individuals with high MeFox and low MMA is biologically plausible. Individuals with competent mitochondria but elevated oxidative stress may be more susceptible to additional damage from glyphosate-induced redox imbalance. In contrast, those with pre-existing mitochondrial dysfunction (i.e., high MMA levels) may exhibit a “ceiling effect”, whereby additional toxic effects become less detectable. However, several alternative explanations may help account for these divergent patterns. Despite statistical adjustment, residual confounding could have influenced the observed associations. Moreover, the limited number of deaths within each stratified subgroup may have contributed to imprecise HR estimates. Given these limitations, the findings should be interpreted with caution and validated in larger, independent cohorts. Overall, our results highlight the complex interplay between glyphosate exposure, mitochondrial function, oxidative stress, folate metabolism, and mortality risk, emphasizing the need for further research to elucidate these interconnected biological pathways.

This study leveraged data from a nationally representative sample of NHANES participants, ensuring broad generalizability to the U.S. population. The use of rigorous, validated methodologies for measuring urinary glyphosate, MeFox, and MMA enhanced the reliability of biomarker assessments. Additionally, the study employed robust statistical methods, including weighted Cox regression models and comprehensive adjustment for potential confounders, to ensure the accurate interpretation of associations. However, a limitation of this study is its observational design, which does not allow for the establishment of a causal relationship. Another limitation is that MeFox and MMA are indirect markers of oxidative stress and mitochondrial impairment. Although both compounds are mechanistically linked to redox imbalance, NHANES 2013–2014 did not assay canonical oxidative-stress endpoints such as lipid peroxidation products, glutathione, or antioxidants. Future population studies integrating these additional biomarkers would allow for a more comprehensive evaluation of glyphosate-related oxidative damage. Additionally, the reliance on single-time-point measurements of biomarkers potentially misses temporal variations. The relatively short follow-up period and limited number of mortality events may have constrained the statistical power for detecting associations with specific mortality outcomes. Furthermore, residual confounding by unmeasured factors and the inability to explore dose–response relationships limit the depth of the findings.

## 5. Conclusions

Following an examination of a representative sample of U.S. adults, this study highlights significant associations between urinary glyphosate levels and biomarkers of oxidative stress and mitochondrial function, specifically MMA and MeFox. While glyphosate exposure was not significantly associated with mortality in the overall population, subgroup analyses revealed elevated all-cause mortality risks in participants with lower MMA levels and higher MeFox levels. The significant interactions between glyphosate and these biomarkers underscore the importance of oxidative stress and mitochondrial function in modulating the health risks associated with glyphosate exposure. These findings suggest the need for further research to clarify the underlying mechanisms and to identify vulnerable populations for targeted public health interventions.

## Figures and Tables

**Table 1:** The geometric means (geometric SE) of urinary glyphosate, MeFox, and MMA in different subgroups.

		Glyphosate (μg/g Creatinine)		MeFox (nmol/L)		MMA (nmol/L)	
	N	Geometric Means (SE)	*p* Value	Geometric Means (SE)	*p* Value	Geometric Means (SE)	*p* Value
Total	1464	0.43 (1.01)		1.61 (1.02)		151.15 (1.01)	
Sex			<0.001		0.155		0.942
Males	707	0.37 (1.03)		1.62 (1.02)		153.96 (1.03)	
Females	757	0.48 (1.03)		1.61 (1.02)		148.58 (1.03)	
Age (years)			<0.001		<0.001		<0.001
19–39	498	0.35 (1.03)		1.39 (1.03)		129.76 (1.02)	
40–59	512	0.43 (1.03)		1.52 (1.03)		147.88 (1.02)	
≥ 60	454	0.54 (1.04)		2.03 (1.04)		183.16 (1.02)	
Ethnicity			<0.001		<0.001		<0.001
Mexican-American	187	0.38 (1.06)		1.30 (1.05)		134.60 (1.04)	
Other Hispanic	128	0.42 (1.07)		1.45 (1.06)		142.74 (1.05)	
Non-Hispanic White	681	0.47 (1.03)		1.87 (1.03)		170.33 (1.02)	
Non-Hispanic Black	261	0.36 (1.05)		1.30 (1.04)		132.80 (1.03)	
Non-Hispanic Asian	164	0.45 (1.06)		1.64 (1.06)		134.15 (1.04)	
Other ethnicity	43	0.36 (1.12)		2.02 (1.10)		154.82 (1.08)	
Smoking status			0.011		0.010		0.337
Non-smoker	893	0.45 (1.03)		1.69 (1.02)		151.50 (1.02)	
ETS	210	0.40 (1.06)		1.52 (1.05)		144.96 (1.03)	
Current smoker	361	0.40 (1.04)		1.50 (1.04)		154.00 (1.02)	
BMI			0.343		<0.001		0.864
<25	440	0.45 (1.04)		1.50 (1.03)		151.33 (1.02)	
25–29	464	0.43 (1.04)		1.51 (1.03)		152.41 (1.02)	
≥ 30	560	0.42 (1.03)		1.80 (1.03)		149.98 (1.02)	
Hypertension			<0.001		<0.001		<0.001
No	848	0.40 (1.03)		1.45 (1.01)		140.34 (1.02)	
Yes	616	0.46 (1.03)		1.87 (1.02)		167.41 (1.03)	
Diabetes Mellitus			<0.001		<0.001		<0.001
No	1228	0.41 (1.02)		1.53 (1.01)		249.15 (1.02)	
Yes	236	0.52 (1.05)		2.14 (1.04)		167.77 (1.05)	
Chronic kidney disease			0.121		<0.001		<0.001
No	1377	0.42 (1.02)		1.54 (1.01)		145.99 (1.02)	
Yes	87	0.48 (1.09)		3.44 (1.06)		261.91 (1.08)	
Hypercholesterolemia			0.015		<0.001		<0.001
No	600	0.40 (1.03)		1.48 (1.02)		141.50 (1.03)	
Yes	864	0.45 (1.03)		1.72 (1.02)		158.24 (1.02)	

Tested by Student’s 2-tailed *t*-test or by one-way analysis of variance. Abbreviations: BMI, body mass index; ETS, environmental tobacco smoke; MeFox, pyrazino-s-triazine derivative of 4-α-hydroxy-5-methyl-tetrahydrofolate; MMA, methylmalonic acid.

**Table 2:** Linear regression coefficients (standard error) of ln-serum MMA and Ln-MeFox oxidation products with a unit increase in ln-urinary glyphosate, ln-serum MeFox, and continuous covariates in multiple linear regression models, with the results weighted for the sampling strategy.

	Ln-MMA (nmol/L)				Ln-MeFox (nmol/L)			
	Model 1		Model 2		Model 1		Model 2	
Continuous Variables	Adjusted *β* (SE)	*p* Value	Adjusted *β* (SE)	*p* Value	Adjusted *β* (SE)	*p* Value	Adjusted *β* (SE)	*p* Value
Urinary glyphosate (Unweighted number/population size = 1464/210267786)								
Ln-glyphosate (µg/L)	0.063 (0.016)	<0.001	0.061 (0.014)	<0.001	0.221 (0.017)	<0.001	0.215 (0.018)	<0.001
Age (years)	0.008 (0.001)	<0.001	0.007 (0.001)	<0.001	0.005 (0.001)	0.003	0.002 (0.002)	0.143
Poverty–income ratio	−0.032 (0.007)	0.001	−0.029 (0.007)	0.001	−0.008 (0.012)	0.525	−0.002 (0.012)	0.881
Ln-urinary creatinine (g/L)	−0.012 (0.020)	0.555	−0.021 (0.018)	0.266	−0.113 (0.030)	0.002	−0.121 (0.032)	0.002
BMI (kg/m^2^)	−0.003 (0.002)	0.239	−0.003 (0.002)	0.207	0.013 (0.002)	<0.001	0.011 (0.003)	0.001
Ln-vitamin B12 (pg/mL)	−0.273 (0.021)	<0.001	−0.284 (0.019)	<0.001	0.050 (0.042)	0.255	0.036 (0.038)	0.355
Serum MeFox * (Unweighted number/population size = 1464/63262637)								
Ln-MeFox (nmol/L)	0.152 (0.018)	<0.001	0.131 (0.017)	<0.001				
Age (years)	0.008 (0.001)	<0.001	0.007 (0.001)	<0.001				
Poverty–income ratio	−0.032 (0.007)	<0.001	−0.030 (0.007)	0.001				
BMI (kg/m^2^)	−0.004 (0.002)	0.047	−0.004 (0.002)	0.049				
Ln-vitamin B12 (pg/mL)	−0.275 (0.019)	<0.001	−0.283 (0.017)	<0.001				

Model 1 adjusted for age, gender, ethnicity, poverty–income ratio, BMI, urinary creatinine, smoking status, drinking status, physical activity, and serum vitamin B12. Model 2 adjusted for Model 1 plus hypertension, diabetes mellitus, chronic kidney disease, hypercholesterolemia. Abbreviations: BMI, body mass index; MeFox, pyrazino-s-triazine derivative of 4-α-hydroxy-5-methyl-tetrahydrofolate; MMA, methylmalonic acid. * Urinary creatinine was not included as a covariate when assessing the association between serum MeFox and serum MMA.

**Table 3:** Linear regression coefficients (SE) of ln-serum MeFox and ln-serum MMA with a unit increase in ln-urinary glyphosate in the subpopulation, with the results weighted for the sampling strategy.

		Ln-MeFox (nmol/L)			Ln-MMA (nmol/L)		
	N	Adjusted *β* (SE)	*p* Value	*p* for Interaction	Adjusted *β* (SE)	*p* Value	*p* for Interaction
Age, years				0.258			0.174
19–49	764	0.203 (0.030)	<0.001		0.078 (0.016)	<0.001	
≥ 50	700	0.237 (0.033)	<0.001		0.048 (0.021)	0.040	
Gender				0.501			0.430
Male	707	0.228 (0.035)	<0.001		0.068 (0.016)	0.001	
Female	757	0.194 (0.035)	<0.001		0.056 (0.019)	0.010	
Ethnicity				0.560			0.010
Non-Hispanic White	681	0.231 (0.027)	<0.001		0.053 (0.025)	0.048	
Other	783	0.191 (0.027)	<0.001		0.077 (0.017)	0.001	
BMI, kg/m^2^				0.550			0.652
< 27.9 (50%ile)	737	0.239 (0.029)	<0.001		0.093 (0.032)	0.010	
≥ 27.9 (50%ile)	727	0.197 (0.031)	<0.001		0.036 (0.020)	0.094	
Hypertension				0.797			0.931
No	848	0.208 (0.035)	<0.001		0.068 (0.020)	0.004	
Yes	616	0.232 (0.043)	<0.001		0.051 (0.021)	0.029	
Diabetes Mellitus				0.494			0.061
No	1228	0.199 (0.025)	<0.001		0.071 (0.015)	<0.001	
Yes	236	0.325 (0.063)	<0.001		0.026 (0.032)	0.431	
Chronic kidney disease				0.543			0.183
No	1377	0.213 (0.018)	<0.001		0.063 (0.016)	<0.001	
Yes	86	0.232 (0.071)	0.006		0.023 (0.043)	0.591	
Hypercholesterolemia				0.575			0.784
No	600	0.187 (0.043)	0.001		0.071 (0.030)	0.031	
Yes	864	0.241 (0.037)	<0.001		0.055 (0.016)	0.003	

Adjusted for Model 2: Age, gender, ethnicity, poverty–income ratio, BMI, urinary creatinine, smoking status, drinking status, physical activity, serum vitamin B12, hypertension, diabetes mellitus, chronic kidney disease, and hypercholesterolemia. Abbreviations: BMI, body mass index; MeFox, pyrazino-s-triazine derivative of 4-α-hydroxy-5-methyl-tetrahydrofolate; MMA, methylmalonic acid.

**Table 4:** HRs (95% CI) for all-cause mortality and cardiovascular mortality associated with a unit increase in ln-glyphosate across different subgroups, derived from a weighted Cox regression model and accounting for the complex sampling design.

Ln-glyphosate (µg/L)	Unweighted No./Population Size	HR	95% CI	*p* Value	*p* for Interaction
All-cause mortality (Adjusted for model 3)					0.773
Total	1463/210,214,570	1.342	0.942—1.912	0.097	
MeFox < 50%ile	734/102,979,608	1.529	0.785—2.978	0.195	
MeFox ≥ 50%ile	729/107,234,961	1.395	1.044—1.864	0.027	
Cardiovascular mortality * (Adjusted for model 3)					0.933
Total	1463/210,214,570	1.207	0.689—2.114	0.486	
MeFox < 50%ile	734/102,979,608	1.410	0.809—2.457	0.208	
MeFox ≥ 50%ile	729/107,234,961	1.367	0.998—1.872	0.051	
All-cause mortality (Adjusted for model 4)					0.002
Total	1463/210,214,570	1.204	0.838—1.729	0.293	
MMA < 50%ile	741/101,870,296	2.679	1.603—4.475	0.001	
MMA ≥ 50%ile	722/108,344,274	0.919	0.645—1.309	0.617	
Cardiovascular mortality * (Adjusted for model 4)					0.038
Total	1463/210,214,570	1.213	0.583—2.526	0.583	
MMA < 50%ile	741/101,870,296	1.689	0.461—6.195	0.403	
MMA ≥ 50%ile	722/108,344,274	1.199	0.709—2.030	0.473	

Model 3: Model 2 plus serum MeFox oxidation product. Model 4: Model 2 plus serum MMA. Abbreviations: HR: hazard ratios; MeFox, pyrazino-s-triazine derivative of 4-α-hydroxy-5-methyl-tetrahydrofolate; MMA, methylmalonic acid. * Cardiovascular mortality: Death from heart or cerebrovascular disease.

## Data Availability

The data used in this study are publicly available from the NHANES database, hosted by the U.S. Centers for Disease Control and Prevention. The datasets analyzed during the current study can be accessed at: https://wwwn.cdc.gov/nchs/nhanes/default.aspx (accessed on 5 April 2025).

## Supplementary Materials

The following supporting information can be downloaded at https://www.mdpi.com/article/10.3390/toxics13050373/s1, Figure S1: Weighted scatter-plot regressions, adjusted for Model 2 covariates, showing the relationships between ln-urinary glyphosate and (A) ln-serum MeFox and (B) ln-serum MMA; Table S1: HR (95% CI) for all-cause mortality and cardiovascular mortality associated with a unit increase in ln-glyphosate across different subgroups of MeFox and MMA, derived from a weighted Cox regression model accounting for complex sampling design.
