# Supplementary material for: Glyphosate Exposure, Oxidative Stress, Mitochondrial Dysfunction, and Mortality Risk in US Adults: Insights from the National Health and Nutrition Examination Survey
Source: Toxics. 2025 May 4;13(5):373. doi: 10.3390/toxics13050373 (PMC12115739; doi:10.3390/toxics13050373)

Supplementary material

Supplemental Table S1. HR (95% CI) for all-cause mortality and cardiovascular mortality associated with a unit increase in ln-glyphosate across different subgroups of MeFox and MMA, derived from a weighted Cox regression model accounting for complex sampling design.

Ln-glyphosate (µg/L)	Unweighted no./population size	HR	95% CI	*P* value	*P* for trend
All-cause mortality					0.577
MeFox < 50%ile and MMA < 50%ile	447/60351099	Reference			
MeFox ≥ 50%ile and MMA < 50%ile	294/41519197	1.044	0.324—3.370	0.938	
MeFox < 50%ile and MMA ≥ 50%ile	287/42628509	1.466	0.622—3.455	0.357	
MeFox ≥ 50%ile and MMA ≥ 50%ile	435/65715764	1.047	0.529—2.071	0.889	
Cardiovascular mortality*					0.581
MeFox < 50%ile and MMA	734/102979608	Reference			

< 50%ile				
MeFox ≥ 50%ile and MMA < 50%ile	729/107234961	1.776	0.419—7.535	0.410
MeFox < 50%ile and MMA ≥ 50%ile	1463/210214570	0.984	0.277—3.499	0.979
MeFox ≥ 50%ile and MMA ≥ 50%ile	741/101870296	1.874	0.425—8.271	0.381

Adjusted for model 2

Abbreviation: HR: Hazard ratios; MeFox, Pyrazino-s-triazine derivative of 4-α-hydroxy-5-methyl-tetrahydrofolate; MMA, Methylmalonic acid.

*Cardiovascular mortality: Death from heart or cerebrovascular disease

Supplemental Figure S1. Weighted scatter-plot regressions, adjusted for Model 2 covariates, showing the relationships between ln-urinary glyphosate and (A) ln-serum MeFox and (B) ln-serum MMA.

A

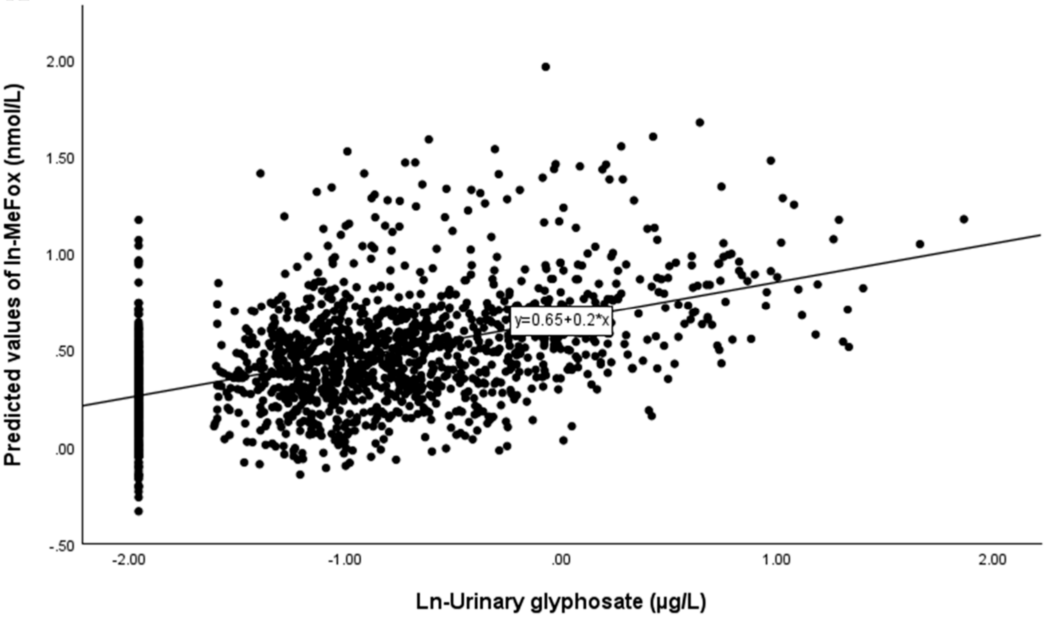

B

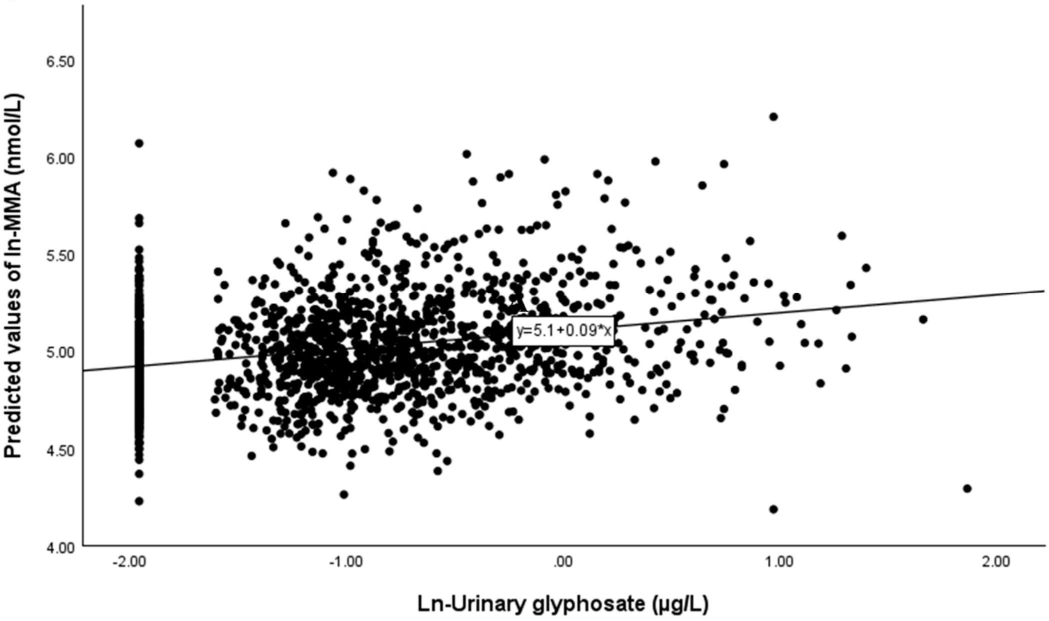

Supplement: Supplementary file 1 [file toxics-13-00373-s001.zip › toxics-3598793-supplementary.pdf]
